# Teaching Cancer Survivors Coping Skills for Managing Fear of Recurrence: Insights From a Pilot Randomized Controlled Trial

**DOI:** 10.1177/27536130251407685

**Published:** 2025-12-21

**Authors:** Aimee J. Christie, Caleb Bolden, Elyse R. Park, Gloria Y. Yeh, Conall O’Cleirigh, Hang Lee, Jeffrey Peppercorn, Lynne I. Wagner, Elisabeth C. Henley, Lara Traeger, Ade Adamson, Anthony D Sung, Daniel L. Hall

**Affiliations:** 1 4002 The University of Texas MD Anderson Cancer Center, Houston, TX, USA; 2 2348 Massachusetts General Hospital, Boston, MA, USA; 3 Harvard Medical School, Boston, MA, USA; 4 1859 Beth Israel Deaconess Medical Center, Boston, MA, USA; 5 Gillings School of Public Health, 2331 University of North Carolina, Chapel Hill, NC, USA; 6 5452 University of Miami, Coral Gables, FL, USA; 7 12330 The University of Texas at Austin, Austin, TX, USA; 8 21638 The University of Kansas Medical Center, Kansas City, KA, USA

**Keywords:** coping, fear of cancer recurrence, cancer survivors, psycho-oncology, intervention, mind-body

## Abstract

**Purpose:**

Fear of cancer recurrence (FCR) is highly common and, if poorly managed, can be distressing and impairing. We developed a virtual, mind-body resiliency intervention for fear of cancer recurrence in survivorship (IN FOCUS), which was shown to be feasible and improved FCR post-intervention. This report aimed to describe coping processes associated with FCR and effects of IN FOCUS on coping over time.

**Method:**

A single-blinded, 2-arm, randomized controlled trial was conducted from July 2021 to March 2022 comparing IN FOCUS (8 weekly, 90-minute, synchronous virtual group classes teaching cognitive behavioral techniques, relaxation training, meditation, adaptive health behaviors, and positive psychology skills) to usual care (synchronous virtual community group support referral) among cancer survivors with non-metastatic disease and clinically elevated FCR (FCR Inventory severity ≥16). Measures included coping styles (Brief COPE) and perceived coping skills (Measure of Current Status-Part A). Intent-to-treat analyses with separate general linear mixed models were used to identify group-by-time effects (Cohen’s *d*; 0.5 a medium effect, 0.8 a large effect) from baseline through 2 months and 5 months.

**Results:**

Sixty-four survivors enrolled (age M = 52 years, time since completing primary cancer treatment M = 5 years). By 5 months, survivors randomized to IN FOCUS (vs usual care) demonstrated multiple effects on coping in the medium to large range. Compared to usual care, IN FOCUS increased problem-focused coping, such as using instrumental support (*d =* 0.60), planning (*d* = 0.60), positive reframing (*d* = 0.48), and active coping (*d* = 0.45). Similarly, IN FOCUS improved emotion-focused coping, specifically venting (*d =* 0.70), acceptance (*d* = 0.58), humor (*d* = 0.50), and religion (*d* = 0.48). IN FOCUS also enhanced survivors’ coping confidence (*d* = 0.79), relaxation skills (*d* = 0.57), and assertiveness (*d* = 0.46). Avoidance-focused coping and awareness of physical tension exhibited less robust changes by 5 months.

**Conclusions:**

Cancer survivors can enhance multiple aspects of coping with FCR through interventions such as IN FOCUS that teach mind-body resiliency techniques.

## Introduction

Roughly 50% of cancer survivors endorse clinically elevated fear of cancer recurrence (FCR).^1^ Defining features of FCR include: high levels of preoccupation, high levels of worry, persistence of worry or fear, and hypervigilance to bodily symptoms for signs of recurrence.^2^ FCR may affect patients in negative ways, such as over-utilization of healthcare services,^3,4^ significant psychological distress and somatic symptoms,^5^ and impairment in functioning.^6^ Furthermore, cancer is a common diagnosis; approximately 40.5% of men and women will be diagnosed with cancer in their lifetimes.^7^ The prevalence of cancer diagnoses, coupled with the high FCR rate, indicates a need to investigate FCR coping strategies. FCR can be conceptualized as a cycle, whereby recurring cues (e.g., scans, cancer events) require recurring coping efforts. If successful, coping efforts will reduce FCR and its negative sequelae, and resiliency is cultivated in the cancer survivor.^8^

There is a robust literature examining the concept of coping in cancer populations showing significant relationships with depression, anxiety, and quality of life.^9^ However, few studies have explored coping mechanisms in the context of FCR. A recent qualitative study examined open-ended survey responses from 347 breast cancer patients enrolled in an FCR randomized controlled trial. Findings indicated that as FCR increased, patients reported using more coping strategies, with a tendency toward avoidant coping.^10^ Overwhelming fear and anxiety triggered attempts to ignore, avoid, or control upsetting emotions and thoughts. Cross-sectional data of 204 Turkish women with breast cancer^11^ reported that higher FCR was associated with the coping orientations of self-punishment and avoidance, while lower FCR was associated with the coping orientation of accommodation (e.g., positive reinterpretation, acceptance, and goal changing). A longitudinal observational study examined coping processes as predictors of FCR in 70 women with newly diagnosed Stage I or II breast cancer.^12^ Results showed that higher avoidance-focused coping was the only significant predictor of higher FCR 1 year later. Coping can mitigate or worsen FCR, emphasizing the importance for FCR interventions to support problem and emotion-focused coping skills.

IN FOCUS is a remotely delivered mind-body intervention for FCR that works to enhance cancer survivors’ ability to manage distressing emotions and address what can be controlled. IN FOCUS builds on previous anxiety interventions by combining multiple therapy modalities, specifically cognitive behavioral therapy techniques (e.g., worry time, identifying and reframing negative automatic thoughts), mind-body skills (e.g., diaphragmatic breathing, meditation, chair yoga, mindfulness), resiliency training (e.g., acceptance, optimism, social support), adaptive health behaviors (diet, exercise, sleep), and positive psychology skills (e.g., gratitude, humor, creativity).^8^ In the pilot randomized controlled trial, 64 adult cancer survivors with elevated FCR were randomly assigned to 8 weekly sessions of synchronous, virtual group (IN FOCUS) or usual care.^13^ IN FOCUS was feasible and acceptable, showed medium-to-large reductions of FCR, and medium-to-large increases in resiliency post-intervention.^13^ However, it is unclear whether IN FOCUS, which teaches participants skills to manage, not lessen, uncertainty, improves coping processes and skills.

This exploratory analysis of IN FOCUS aimed to (1) describe the baseline means for FCR-related coping (ie, coping responses and self-perceived coping ability) and (2) explore group-by-time effects of these coping-related constructs across 5 months. Findings from the current study will elucidate how coping and perceptions of coping ability change in response to IN FOCUS and will expand our understanding of FCR management. Understanding how FCR interventions influence specific coping strategies and perceptions of coping ability may provide a unique opportunity to understand mechanisms of change for future intervention studies.

## Methods

### Participants, Randomization, and Design

Design, recruitment, and participant details have been previously described.^13^ Eligibility criteria included: (1) adult cancer survivors who had completed treatment with curative intent (ie, surgery, chemo, and/or radiation) for any non-metastatic, localized, or regional solid or blood malignancies with (2) elevated FCR (FCR Inventory severity≥16). Ineligible criteria included FCR Inventory severity<16, metastatic cancer, difficulties speaking and writing in English, untreated serious mental illness, psychiatric hospitalization in the past year, and active suicidal ideation. After completing the baseline survey, survivors were provided unique study ID numbers and randomized (1:1) to either IN FOCUS or usual care. Randomization was overseen by a biostatistician (HL). One non-transparent bag contained 64 uniform slips of paper labeled with study IDs, shaken repeatedly to randomize. A separate non-transparent bag contained 10 uniform slips of paper labeled with a study arm (1:1 intervention or control). For each participant randomized, in the presence of an observer, one slip was drawn from each bag and recorded. The principal investigators and biostatistician were blinded and only had access to participant study IDs and treatment arm. Survivors were not blinded to their allocation, because those randomized to usual care were referred to cancer support groups and those randomized to IN FOCUS were not. Patient-reported outcomes on psychological measures were collected at 3 timepoints: baseline (T0), 2 months/post-intervention (T1), and 5 months/3 months post-intervention (T2).

#### Intervention for Fear of Cancer Recurrence and Uncertainty in Survivorship (IN FOCUS)

IN FOCUS content and delivery plans have been refined through multiphase testing using the ORBIT framework.^8,14^ Modeled after the Stress Management and Resiliency Training - Relaxation Response Resiliency Program (SMART-3RP),^15,16^ groups were comprised of 6-8 survivors and met in 8, weekly, 90-minute sessions delivered via Zoom. IN FOCUS sessions were facilitated by a psychiatrist who specialized in oncology treatment and previously completed certification training in the SMART-3RP. Weekly supervision was provided by a different psychiatrist with expertise in the SMART-3RP. Session instructions included three core areas: mind-body techniques to elicit the relaxation response, cognitive behavioral techniques to identify and interrupt patterns underlying worry and avoidance, and positive psychology techniques to foster humor, compassion, and empathy. In addition to these skills, three sessions included health education on healthy eating, sleep, and physical activity, informed by 2019 NCCN cancer survivorship guidelines.^17^ This resiliency treatment approach is multimodal and emphasizes the interplay between physiological responses to stress (and its counterpoint, the relaxation response), cognitive-behavioral factors, social support, health behaviors, and positive psychology.

#### Usual Care (UC)

UC content and delivery plans have been previously described,^8^ and consisted of providing a referral for synchronous, virtual cancer support group sessions, matched in session duration to IN FOCUS. Groups sessions were 90 minutes and co-facilitated by a trained clinician (social worker, psychologist, psychiatrist, nurse, nurse practitioner, physician assistant, or primary care physician) and a cancer survivor peer. Content was unstructured and did not include training in skills covered in IN FOCUS.

### Measures

#### Sociodemographic, Psychiatric and Cancer Characteristics

Sociodemographic characteristics were self-reported. Medical characteristics were self-reported and verified by medical record, including date(s) of cancer diagnoses, cancer treatment history, date(s) of primary cancer treatment completion, physical and psychiatric co-morbidities, and current medication use. A systematic coding process was conducted to verify participant health information using electronic health records.

#### Outcome Measures (T0, T1, T2)

##### Brief – Coping Orientation to Problems Experienced Inventory (B-COPE)

The B-COPE is a 28-item measure assessing how individuals cope with adversity^18^ and has been validated in heterogeneous cancer survivor samples.^9,19^ The B-COPE contains 14 scales that assess responses to stressors, each with a specific conceptual focus: (a) acceptance; (b) active coping, (c) behavioral disengagement, (d) denial, (e) humor, (f) planning, (g) positive reframing, (h) religion, (i) self-blame, (j) self-distraction, (k) substance use, (l) using emotional support, (m) using instrumental support, and (n) venting. Instructions for the B-COPE in this study ask participants what they have been doing to cope with fear about the possibility of cancer recurring. Item responses were *I didn’t do this at all* = 0, *I did this a little bit* = 1, *I did this a medium amount* = 2, and *I did this a lot* = 3. Each subscale is measured using 2 items, which are summed (possible range 0-6). Higher scores indicate more frequent use of that specific coping response. Subscale scores were analyzed independently.

Some research has reported that the individual coping responses reflect overarching coping styles: problem-focused coping (e.g.*, I've been taking action to try to make the situation better*), emotion-focused coping (e.g., *I've been expressing my negative feelings)*, and avoidance-focused coping (e.g.*, I've been saying to myself “this isn't real”)*.^20^ In the context of the B-COPE and for the purposes of the present study, problem-focused coping includes the subscales of active coping, use of instrumental support, positive reframing, and planning. Emotion-focused coping includes the subscales of emotional support, venting, humor, acceptance, religion, and self-blame. Avoidance-focused coping includes the subscales of self-distraction, denial, substance use, and behavioral disengagement. For the purpose of interpretation and readability, subscale results will be organized by overarching coping style.

##### Measure of Current Status-A (MOCS-A)

The MOCS-A is a 13-item measure assessing self-perception of skills after participating in an intervention^21^ and has been validated in cancer survivors.^22-24^ Instructions ask participants to indicate how well one can currently practice the identified skill, or how confident one is in being able to do each skill. The specific skills, measured by subscales of the MOCS-A, include assertiveness to address needs (3 items; e.g.*, I can clearly express my needs to other people who are important to me*), coping confidence (5 items; e.g.*, When problems arise I know how to cope with them*), relaxation skills (2 items; e.g.*, I am able to use mental imagery to reduce any tension I experience*), and the ability to perceive physical signs of tension (3 items; e.g.*, I become aware of my tightness in my body as soon as it develops*). Items are rated on a 5-point Likert scale from strongly disagree = 0 to strongly agree = 4 and summed to create subscale scores. Higher scores indicate stronger perceived skills.

##### Fear of Cancer Recurrence – Short Form (FCRI-SF)

The FCRI-SF is a 9-item measure (e.g., *I am afraid of cancer recurrence*) assessing the frequency and severity of thoughts relating to cancer recurrence^25^ and validated in heterogeneous cancer samples.^1,26^ Items are rated on a 5-point Likert scale with higher scores indicating greater fear of cancer recurrence, and possible total score range is 0-36. Scores 16-21 indicate elevated FCR, and scores ≥22 indicate clinically elevated FCR.^27^ Group-by-time changes in FCR have been previously reported.^13^

### Statistical Analysis

Analyses were conducted with de-identified data using SPSS under the guidance of a biostatistician (HL). All variables were examined for normal distributions. Pearson correlation coefficients were calculated to examine relationships between measures at T0. Quantitative outcomes were analyzed separately using intent-to-treat with separate general linear mixed models to identify preliminary effects of the intervention across all 3 timepoints (T0-T2) with separate contrasts from T0 through T1 and T2. Across models, time was treated as a 3-level categorical variable (T0, T1, T2) to obtain estimated marginal means and 95% confidence intervals by condition at each timepoint. Models included T0 as the timepoint reference and UC as the treatment group reference to produce separate contrasts between T0-T1 and T0-T2 for IN FOCUS vs UC. Missing data were handled using the maximum likelihood which incorporated information from all randomized participants. To estimate magnitudes of any observed pre-post intervention changes, Cohen’s *d* was calculated using the mixed model-based estimated between-group difference in changes from T0 over the specified time period, divided by the observed, model-free standard deviation of the change from T0 over the specified time period among the entire sample (*d* = 0.3 indicates a small effect, 0.5 a medium effect, and 0.8 a large effect). *P*-values were not computed, as the pilot RCT was not powered to detect a statistically significant difference in its primary (feasibility) outcomes.

## Results

Patient characteristics have been detailed previously^13^ and are displayed in Supplemental Table 1. Participants were primarily female (83%), white (92%), with a mean age of 52 years. Mean time since diagnosis was 8.3 years and mean time since treatment was 5 years. The top three cancer diagnoses were breast (45%), hematological (17%), and genitourinary (13%). At T0, the mean FCR severity was 22.92 (SD = 4.57). Correlations between FCR and B-COPE scales and MOCS-A scales are reported in Table 1. There were no significant correlations between FCR and coping scales or perceptions of coping ability at baseline (*p* ≥ .05). Means and standard deviations for coping skills, as measured by the B-COPE, are displayed in Table 1. At baseline, there were no differences in coping responses in the IN FOCUS and UC groups with both reporting that the most frequently used coping responses were acceptance (emotion-focused), self-distraction (avoidance-focused), and active coping (problem-focused). The most infrequently used coping responses used by both groups were denial (avoidance-focused), substance use (avoidance-focused), and behavioral disengagement (avoidance-focused). Means and standard deviations for perceptions of coping ability, as measured by the MOCS-A, are displayed in Table 1. At baseline, both IN FOCUS and UC groups reported similar levels of coping confidence, awareness of tension, assertiveness, and relaxation.

**Table 1.** Fear of Cancer Recurrence and Coping Zero-Order Correlations

Variable	M (SD); observed range	*r*	*p*
FCR	22.92 (4.57); 13-35		
Problem-focused coping			
Active coping	3.76 (1.41); 1-6	0.09	0.46
Planning	3.29 (1.65) 1-6	0.25	0.05
Positive reframing	3.02 (1.44); 1-6	0.06	0.66
Use of instrumental support	3.23 (1.83); 1-6	0.19	0.13
Emotion-focused coping			
Acceptance	4.44 (1.31); 1-6	−0.16	0.23
Humor	1.64 (1.75); 1-6	0.10	0.42
Religion	2.58 (2.15); 1-6	−0.05	0.68
Self-blame	1.88 (1.63); 1-6	0.18	0.15
Use of emotional support	3.22 (1.77); 1-6	0.07	0.60
Venting	2.16 (1.42); 1-5	0.13	0.33
Avoidance-focused coping			
Behavioral disengagement	0.73 (1.36); 1-4	0.04	0.75
Denial	0.55 (0.94); 1-6	0.20	0.12
Self-distracting	3.91 (1.55); 1-6	0.06	0.62
Substance use	0.67 (1.37); 1-6	0.19	0.13
Perceptions of coping ability			
Assertiveness	6.78 (2.95); 1-12	−0.06	0.64
Coping confidence	10.00 (3.61); 2-20	−0.15	0.25
Relaxation	3.21 (1.93); 1-8	−0.18	0.17
Tension awareness	7.14 (2.75); 2-12	−0.10	0.43

*Note.* N = 64; FCR = Fear of cancer recurrence. *r* = Pearson’s correlation.

### Problem-Focused Coping

**Figure 1.**
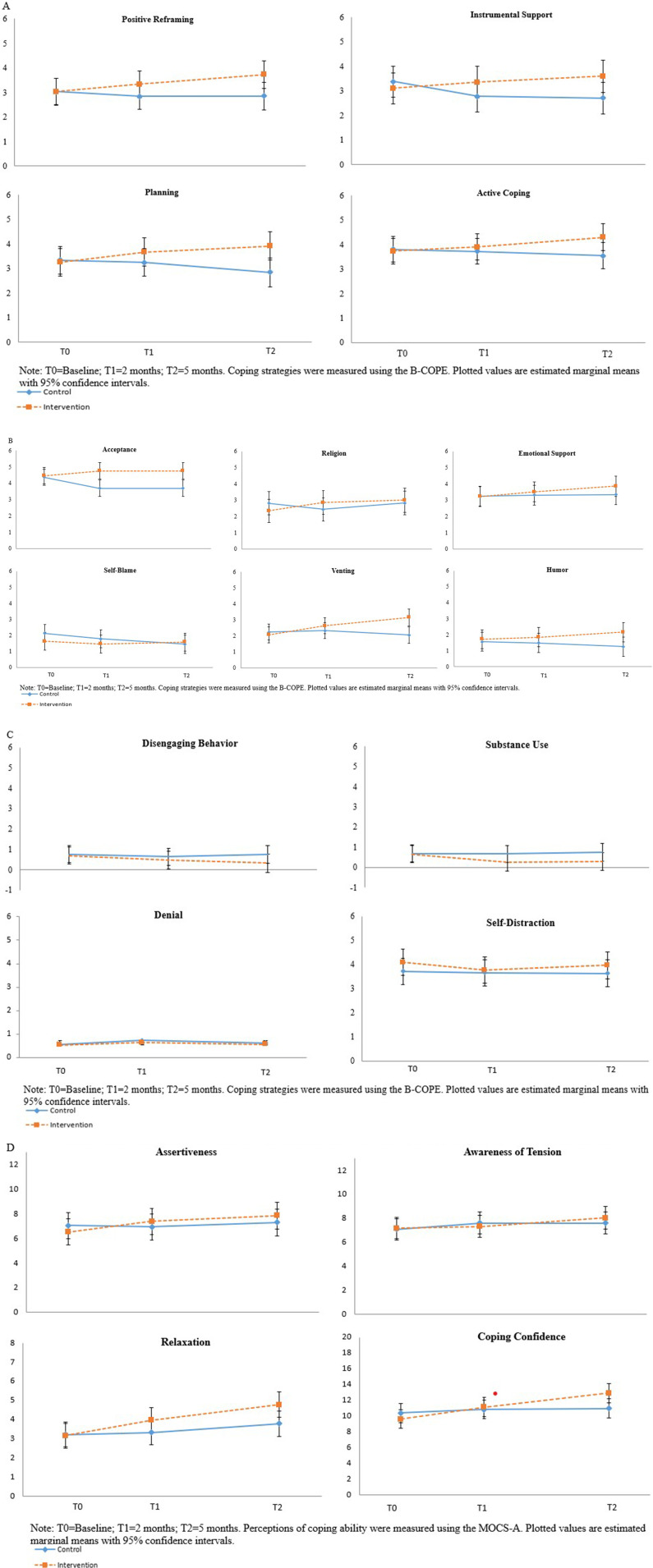
(A) Linear mixed models of problem-focused coping among cancer survivors randomized to IN FOCUS vs usual care. (B) Linear mixed models of emotion-focused coping among cancer survivors randomized to IN FOCUS vs usual care. (C) Linear mixed models of avoidance-focused coping among cancer survivors randomized to IN FOCUS vs usual care. (D) Linear mixed models of perceptions of coping ability among cancer survivors randomized to IN FOCUS vs usual care

As shown in Table 2 and Figure 1A, by 2 months, IN FOCUS (vs UC) showed small effect size increases in problem-focused coping in terms of use of instrumental support (*d* = 0.48) and positive reframing (*d* = 0.30). By 5 months, these group-by-time effects had increased in all problem-focused coping domains: planning (*d* = 0.60), use of instrumental support (*d* = 0.60), positive reframing (*d* = 0.48), and active coping (*d* = 0.45).

**Table 2.** Coping Skills Estimated Marginal Means From Baseline to 2 Months and 5 Months

	Time	IN FOCUS M (95% CI)	Usual care M (95% CI)	Slope difference M (95% CI)	SD of ΔT0 under H_0_	*d*
Problem-Focused Coping						
Active coping	T0	3.72 (3.20 to 4.23)	3.79 (3.27 to 4.31)			
	T1	3.89 (3.36 to 4.42)	3.71 (3.19 to 4.23)	0.24 (−0.57 to 1.05)	1.58	0.15
	T2	4.29 (3.74 to 4.83)	3.53 (2.99 to 4.06)	0.83 (−0.00 to 1.66)	1.84	0.45
Planning	T0	3.25 (2.69 to 3.81)	3.33 (2.77 to 3.89)			
	T1	3.66 (3.09 to 4.23)	3.24 (2.68 to 3.81)	0.50 (−0.41 to 1.40)	1.99	0.25
	T2	3.91 (3.33 to 4.50)	2.84 (2.26 to 3.41)	1.16 (0.23 to 2.09)	1.94	**0.60**
Positive reframing	T0	3.03 (2.50 to 3.57)	3.02 (2.48 to 3.56)			
	T1	3.33 (2.78 to 3.88)	2.84 (2.30 to 3.38)	0.48 (−0.34 to 1.30)	1.66	0.30
	T2	3.72 (3.16 to 4.28)	2.84 (2.29 to 3.40)	0.87 (0.03 to 1.71)	1.83	0.48
Use of instrumental support	T0	3.09 (2.47 to 3.72)	3.38 (2.75 to 4.00)			
	T1	3.35 (2.71 to 3.99)	2.77 (2.14 to 3.40)	0.86 (0.01 to 1.72)	1.81	0.48
	T2	3.60 (2.93 to 4.26)	2.70 (2.06 to 3.35)	1.17 (0.29 to 2.06)	1.96	**0.60**
Emotion-focused coping						
Acceptance	T0	4.47 (3.97 to 4.96)	4.37 (3.88 to 4.87)			
	T1	4.76 (4.26 to 5.27)	3.70 (3.20 to 4.19)	0.97 (0.31 to 1.64)	1.43	**0.68**
	T2	4.76 (4.26 to 5.27)	3.70 (3.20 to 4.19)	0.97 (0.31 to 1.64)	1.67	**0.58**
Humor	T0	1.72 (1.13 to 2.31)	1.56 (0.98 to 2.15)			
	T1	1.84 (1.25 to 2.44)	1.49 (0.89 to 2.08)	0.20 (−0.57 to 0.97)	1.61	0.12
	T2	2.16 (1.55 to 2.77)	1.25 (0.65 to 1.86)	0.75 (−0.03 to 1.54)	1.56	**0.50**
Religion	T0	2.34 (1.63 to 3.06)	2.81 (2.10 to 3.53)			
	T1	2.85 (2.13 to 3.58)	2.43 (1.71 to 3.15)	0.89 (0.21 to 1.57)	1.41	**0.63**
	T2	3.00 (2.27 to 3.73)	2.82 (2.09 to 3.55)	0.65 (−0.05 to 1.35)	1.36	0.48
Self-blame	T0	1.63 (1.07 to 2.18)	2.13 (1.57 to 2.68)			
	T1	1.46 (0.90 to 2.02)	1.78 (1.21 to 2.34)	0.18 (−0.51 to 0.88)	1.58	0.11
	T2	1.57 (1.00 to 2.15)	1.44 (0.88 to 2.01)	0.63 (−0.07 to 1.33)	1.48	0.43
Use of emotional support	T0	3.22 (2.62 to 3.82)	3.24 (2.64 to 3.85)			
	T1	3.51 (2.90 to 4.13)	3.31 (2.70 to 3.92)	0.23 (−0.46 to 0.92)	1.44	0.20
	T2	3.86 (3.24 to 4.49)	3.34 (2.72 to 3.96)	0.55 (−0.16 to 1.26)	1.40	0.40
Venting	T0	2.08 (1.58 to 2.57)	2.25 (1.75 to 2.74)			
	T1	2.63 (2.13 to 3.13)	2.33 (1.83 to 2.83)	0.47 (−0.33 to 1.27)	1.65	0.28
	T2	3.16 (2.63 to 3.68)	2.06 (1.55 to 2.57)	1.27 (0.44 to 2.09)	1.81	**0.70**
Avoidance-focused coping						
Behavioral disengagement	T0	0.69 (0.27 to 1.11)	0.76 (0.34 to 1.19)			
	T1	0.47 (0.04 to 0.90)	0.63 (0.21 to 1.06)	−0.09 (−0.73 to 0.55)	1.33	−0.07
	T2	0.31 (−0.13 to 0.76)	0.75 (0.32 to 1.19)	−0.37 (−1.02 to 0.29)	1.21	−0.31
Denial	T0	0.53 (0.15 to 0.91)	0.56 (0.18 to 0.94)			
	T1	0.64 (0.25 to 1.03)	0.73 (0.34 to 1.12)	−0.06 (−0.57 to 0.46)	0.89	−0.07
	T2	0.57 (0.17 to 0.96)	0.62 (0.23 to 1.02)	−0.03 (−0.54 to 0.49)	1.07	−0.03
Self-distraction	T0	4.09 (3.56 to 4.63)	3.72 (3.18 to 4.26)			
	T1	3.77 (3.22 to 4.32)	3.67 (3.12 to 4.21)	−0.23 (−1.11 to 0.56)	1.64	−0.14
	T2	3.97 (3.41 to 4.54)	3.64 (3.08 to 4.19)	−0.04 (−0.90 to 0.81)	1.83	−0.02
Substance use	T0	0.66 (0.24 to 1.08)	0.69 (0.27 to 1.11)			
	T1	0.24 (−0.19 to 0.67)	0.67 (0.25 to 1.10)	−0.40 (−0.90 to 0.09)	1.03	−0.39
	T2	0.28 (−0.16 to 0.71)	0.75 (0.32 to 1.18)	−0.44 (−0.95 to 0.07)	1.18	−0.37

*Note.* T0 = Baseline; T1 = 2 months; T2 = 5 months. M = estimated marginal mean; MD = mean difference; CI = Confidence Interval; *d* was calculated using the mixed model-based estimated between-group difference in changes from T0 over the specified time period, divided by the observed, model-free standard deviation of the change from T0 over the specified time period among the entire sample. Bolded items indicate medium and large effect sizes (*d* ≥ 0.5).

### Emotion-Focused Coping

As shown in Table 2/Figure 1B, by 2 months, IN FOCUS (vs UC) produced a medium effect size increase in acceptance (*d* = 0.68) and religion (*d* = 0.63). By 5 months (T2), IN FOCUS produced medium effects for increases in 3 of the emotion-focused coping responses: venting (*d* = 0.70), acceptance (*d* = 0.58), and humor (*d* = 0.50), and small effects for increases in the use of religion (*d* = 0.48) and emotional support (*d* = 0.40). In the UC arm, self-blame scores had a slight reduction by 5 months, while self-blame did not change in the IN FOCUS arm (*d* = 0.43). Notably, this effect size was similar in magnitude to a difference in self-blame scores observed at baseline.

### Avoidance-Focused Coping

As shown in Table 2/Figure 1C, by 2 months, IN FOCUS (vs UC) produced a small effect for reductions in substance use (*d* = −0.39). By 5 months, IN FOCUS (vs UC) reduced avoidance-focused coping to a small effect for substance use (*d* = −0.37) and behavioral disengagement (*d* = −0.31). Neither arm influenced denial (*d* = −0.03) or self-distraction (*d* = −0.02) by 5 months.

### Perceptions of Coping Ability

As shown in Table 3 and Figure 1D, by 2 months, IN FOCUS (vs UC) increased relaxation (*d* = 0.41), perceived assertiveness (*d* = 0.41), and coping confidence (*d* = 0.32) to a small-sized effect. Tension awareness had increased in UC (vs IN FOCUS) by 2 months (*d* = −0.12). By 5 months, IN FOCUS (vs UC) produced medium increases in coping confidence (*d* = 0.79), relaxation (*d* = 0.57), and assertiveness (*d* = 0.46). By 5 months, tension awareness had increased more in IN FOCUS (vs UC), (*d* = 0.10).

**Table 3.** Perceptions of Coping Ability Estimated Marginal Means From Baseline to 2 months and 5 months

	Time	IN FOCUS M (95% CI)	Usual care M (95% CI)	Slope difference M (95% CI)	SD of ΔT0 under H_0_	*d*
Assertiveness	**T0**	6.53 (5.48 to 7.59)	7.06 (5.99 to 8.12)			
	**T1**	7.40 (6.33 to 8.47)	6.96 (5.89 to 8.02)	0.97 (−0.13 to 2.06)	2.37	0.41
	**T2**	7.88 (6.80 to 8.97)	7.31 (6.24 to 8.39)	1.10 (−0.03 to 2.22)	2.38	0.46
Coping confidence	**T0**	9.63 (8.43 to 10.82)	10.38 (9.18 to 11.57)			
	**T1**	11.14 (9.91 to 12.37)	10.84 (9.64 12.05)	1.05 (−0.47 to 2.57)	3.24	0.32
	**T2**	12.90 (11.66 to 14.14)	10.96 (9.73 to 12.18)	2.69 (1.15 to 4.24)	3.41	**0.79**
Relaxation	**T0**	3.15 (2.51 to 3.8)	3.21 (2.56 to 3.86)			
	**T1**	3.97 (3.31 to 4.62)	3.33 (2.67 to 3.98)	0.70 (−0.15 to 1.55)	1.71	0.41
	**T2**	4.77 (4.11 to 5.44)	3.78 (3.11 to 4.45)	1.05 (0.18 to 1.92)	1.83	**0.57**
Tension awareness	**T0**	7.19 (6.29 to 8.09)	7.09 (6.19 to 7.99)			
	**T1**	7.32 (6.40 to 8.24)	7.62 (6.71 to 8.53)	−0.39 (−1.58 to 0.80)	3.26	−0.12
	**T2**	8.05 (7.11 to 8.98)	7.62 (6.68 to 8.56)	0.33 (−0.89 to 1.56)	3.26	0.10

*Note.* T0 = Baseline; T1 = 2 months; T2 = 5 months. M *=* estimated marginal mean; CI = Confidence Interval; *d* was calculated using the mixed model-based estimated between-group difference in changes from T0 over the specified time period, divided by the observed, model-free standard deviation of the change from T0 over the specified time period among the entire sample. Bolded items indicate medium and large effect sizes (*d* ≥ 0.5).

## Discussion

There is a paradox of FCR, such that even after completing cancer treatment with curative intent, there is a need to continually confront fear throughout survivorship. This is due to diverse risks of recurrence across cancer types, repeated cancer screening, as well as continual vigilance for physical symptoms. Often FCR persists even with reports documenting no evidence of disease. IN FOCUS is a multimodal mind-body intervention, which has been shown to decrease FCR and increase resiliency.^13^ The present study details exploratory outcomes of IN FOCUS and aims to describe coping responses at baseline, as well as evaluate changes in coping outcomes in response to IN FOCUS vs usual care. Exploratory analyses allow us to “look under the hood” and examine potential mechanisms of change. Exit interviews with participants spoke of the importance of new coping skills and the resiliency training.^13^ Indeed, the current study expands on that feedback by using a validated measure of coping and of perceptions of coping ability to examine active ingredients of the intervention.

Prior to starting the intervention, participants most frequently used acceptance (emotion-focused), self-distraction (avoidance-focused), and active coping (problem-focused). The three most infrequently used coping responses were denial, substance use, and behavioral disengagement, which were all considered avoidance-focused. In contrast to previous reports suggesting that avoidance-focused coping is positively associated with FCR,^10-12^ the present study identified no baseline associations between any of the measured coping styles and FCR. Moreover, avoidance-focused coping was, on average, low in our sample of survivors with elevated FCR. One explanation may be that people interested in enrolling in a study to manage FCR may be less likely to engage in avoidance-focused strategies. In other words, enrolling in an FCR trial requires effort to confront one’s fear. Alternatively, the present sample represented a wide range of time since completion of cancer treatment. Coping responses may be dynamic as individuals progress through survivorship. Five years is often considered an important inflection point for cancer patients, such that no evidence of disease may get the distinction of one being “cured.”

Self-distraction was the only avoidance-focused coping response that was not low at baseline; in fact, it was in the top three coping responses. Data from the present study suggest that self-distraction may be capturing something distinct from substance use, denial, or behavioral disengagement (ie, “giving up trying to cope”). The self-distraction items are “I’ve been turning to work or other activities to take my mind off things” and “I’ve been doing something to think about it less, such as going to movies, watching TV, reading, daydreaming, sleeping, or shopping.” Perhaps self-distraction does not indicate avoidance-focused coping, but rather a reflection of using pleasurable and meaningful activities for improved mood, termed behavioral activation. If this is the case, self-distraction would be likely considered an emotion-focused coping response. Cancer survivors may be effectively responding to a future that is outside of their control by focusing on the present moment and enjoying activities like reading or watching movies. Future research could utilize qualitative data to help to clarify how self-distraction is subjectively experienced.

In general, IN FOCUS findings suggest an improvement in problem and emotion-focused coping skills. The largest effect size changes were in venting, planning, use of instrumental support, and acceptance. Group-delivered interventions, such as IN FOCUS, enable participants to express negative feelings (ie, vent) and receive validation and advice (ie, use of instrumental support) from other cancer survivors. Additionally, IN FOCUS session content utilized a resiliency-informed framework to highlight the need for acceptance of the unknown and plan for the future even when health outcomes cannot be fully predicted. Central to the IN FOCUS program was its incorporation of mind-body techniques that emphasized relaxation, appreciating the small things in life, social connection, and focusing on the present moment rather than focusing on negative future scenarios, all of which aimed to increase acceptance of uncertainty.^8^ The planning strategies included throughout IN FOCUS, such as engaging in health behaviors, may have allowed survivors to have a sense of control over their health and future, instilling a renewed sense of purpose in their life. Therefore, broadly speaking, improvements in emotion-focused and problem-focused coping is likely related to how IN FOCUS emphasized the positive aspects of life. IN FOCUS’s positive psychology framework encouraged creating adaptive behaviors so cancer survivors may engage with life and their feelings, rather than avoid them. Although levels of self-blame did not change over time in IN FOCUS, survivors in the usual care arm experienced a modest reduction. We interpret this effect with caution. Given the low pooled mean at baseline (1.9 out of 6), a potential floor effect may explain why the small differences at baseline disappeared by month 5. It is also possible that self-blame may require other intervention approaches not taught in IN FOCUS, such as trauma-centered therapies, self-compassion therapies, and acceptance-based therapies.

IN FOCUS findings also suggest reduced avoidance-focused coping, specifically substance use and behavioral disengagement. These changes are surprising given how infrequently participants engaged in substance use and behavioral disengagement at baseline. IN FOCUS may have influenced substance use and behavioral disengagement by enhancing their capacity to tolerate distress, appreciate day-to-day life, and engage in healthy lifestyle behaviors focused on sleep, nutrition, and physical activity. IN FOCUS results suggest no effect on denial or self-distraction. Means at baseline for denial were very low and the lowest of all the coping responses, suggesting a possible floor effect. The lack of change in self-distraction may be related to the concerns addressed earlier in this discussion regarding whether self-distraction reflects avoidance or rather behavioral activation. Managing FCR may need a nuanced understanding of coping responses to identify what is helpful for cancer survivors.

The current study also adds to the literature by documenting the changes in perceptions of coping ability after engaging in a mind-body intervention. The MOCS-A, which was used to measure perceptions of coping ability, was designed to assess the source of an intervention’s beneficial effects, in part to identify the “active ingredients” in an intervention. Participants from the current study perceived improvements in their ability to cope, relax, and be assertive. More than 8 relaxation skills were taught during IN FOCUS, and participants were encouraged to practice at least one of these daily. Improvements in assertiveness may have stemmed from survivors gaining clarity on controllable vs uncontrollable aspects of uncertainty, a key focus of Session 7. The multiple improvements in perceptions suggest that IN FOCUS is working through several pathways, rather than just one. Notably, awareness of physical tension did not change in the present sample, suggesting this capacity may require more skills on mindful awareness of tension than is taught in IN FOCUS. Future studies should continue to examine perceptions of coping ability to understand how interventions may be affecting these outcomes.

An interesting pattern of results emerged, suggesting that coping strategies and perceptions may continue to improve even after the intervention was completed, transitioning from primarily small effects at month 2 to primarily medium effects by month 5. This pattern of effects increasing over time is similar to the trend seen in measures of resilience in the same sample.^13^ It is possible this pattern is a consequence of the confluence of perceived benefit, tool adherence, and habit formation. In fact, IN FOCUS had the largest effect on improving coping confidence. The combination of these results (increases in problem and emotion focused coping + coping confidence) are particularly meaningful, as qualitative research has found high FCR to be associated with more avoidant coping and lower perceived effectiveness of coping.^10^ Early evidence suggests that IN FOCUS directly targets these concerns in cancer survivors.

Our findings contribute to the small qualitative and quantitative literature of FCR and coping in patients with breast cancer.^10-12^ A strength of the present study is the sample, which includes a range of cancer diagnoses and stages, thus improving generalizability. Our study also examines FCR intervention outcomes and demonstrates that coping is indeed malleable in the face of high FCR. IN FOCUS offers several unique benefits compared to other interventions, such as being delivered synchronously in a group to maximize social support and virtually to increase remote access to care. Previous research has demonstrated that cancer survivors with avoidance-focused coping can still engage effectively in FCR interventions, particularly if skills are taught remotely.^28^

Outcomes from the present study compare similarly to one other intervention study. Fear of Cancer Recurrence Therapy (FORT), an FCR intervention using a cognitive existential approach, examined three outcomes from the B-COPE scale: positive reframing, emotional support, and acceptance.^29^ The intervention showed medium effects for increasing acceptance, with significant between group differences 3 months post-treatment. There were also some similarities in the pattern of effects between IN FOCUS and FORT, with effect sizes that increased over time.^29^ This early work in the field of intervention research suggests that targeting coping responses may be worth exploring as a mechanism for reducing FCR. Alternatively, given that the primary outcomes of IN FOCUS showed the largest changes in FCR at month 2,^13^ one possibility is that reducing FCR enabled participants to continue to improve coping responses by month 5. In other words, reducing the clinically significant negative emotion provided increased bandwidth to explore more helpful coping responses. Additional research with larger samples is needed to replicate and test this possibility.

## Limitations

The present study examined exploratory outcomes from an RCT of a mind-body intervention for FCR. Results are exploratory in nature and help to provide directions for future research; thus, we did not control for multiple comparisons. Additionally, the study was not powered to examine significant differences between groups. Reporting effect size changes can direct future research on promising targets for treatment. The study sample consisted of mostly white, non-Hispanic, college-educated women, which limits generalizability to other races, ethnicities, and men. Overall, more research is needed on FCR among racial and ethnic minorities or survivors living with advanced cancer or undergoing treatment.^30,31^ In addition, time since last treatment was highly variable and may account for some variance in FCR. Lastly, this trial did not include long-term assessments beyond 5 months, which may be needed to observe patterns of FCR, coping, and resilience as survivors navigate cancer-related events.

## Future Directions

Future studies should focus on ways to simultaneously improve problem and emotion-focused coping while reducing avoidance-focused coping. Targeting the first 5 years after treatment, where coping may be more variable in the context of FCR, may provide more beneficial effects of treatment. Future studies could also incorporate measures of other life stressors and cancer-related stressors (e.g., timing of scans). Qualitative data has suggested that as FCR increases, perceived effectiveness of coping decreases^10^; future studies could include measures of perceived effectiveness of coping. Previous research of teaching meditation has suggested that positive emotion may predict who continues practicing intervention skills months after completing an intervention.^32^ In fact, IN FOCUS highlighted skills that may foster positive emotions, such as humor, creativity, empathy, and self-compassion. Perhaps future studies with larger sample sizes of IN FOCUS groups can test for how positive emotions may moderate or mediate changes in coping and FCR. In addition, the field should consider novel interventions for patients with metastatic disease to address fear of cancer progression. Future studies could examine how coping responses change in response to an intervention if cancer is more advanced. Finally, future efficacy studies could consider an active skills-based intervention comparison group instead of a usual care arm for more effective patient blinding.

## Conclusions

In sum, IN FOCUS was found to improve problem and emotion-focused coping strategies and perceptions of coping ability, while reducing avoidance-focused coping strategies. We found improvements in both problem and emotion-focused coping strategies, reflecting IN FOCUS’s goal of focusing on positive life characteristics. Additionally, IN FOCUS’s emphasis on relaxation skills resulted in the improvement of perceptions of coping ability. Future research would benefit from studying interventions to improve problem and emotion-focused coping strategies while reducing avoidance-focused strategies among cancer survivors who experience high FCR.

## Supplemental Material

Supplemental Material - Teaching Cancer Survivors Coping Skills for Managing Fear of Recurrence: Insights From a Pilot Randomized Controlled Trial Supplemental Material for Teaching Cancer Survivors Coping Skills for Managing Fear of Recurrence: Insights From a Pilot Randomized Controlled Trial by Aimee J. Christie, Caleb Bolden, Elyse R. Park, Gloria Y. Yeh, Conall O’Cleirigh, Hang Lee, Jeffrey Peppercorn, Lynne I. Wagner, Elisabeth C. Henley, Lara Traeger, Ade Adamson, Anthony Sung, Daniel L. Hall in Global Advances in Integrative Medicine and Health

## Data Availability

The data that support the findings of this study are available from the corresponding author upon reasonable request.*
