# Supplementary material for: Teaching Cancer Survivors Coping Skills for Managing Fear of Recurrence: Insights From a Pilot Randomized Controlled Trial
Source: Glob Adv Integr Med Health. 2025 Dec 21;14:27536130251407685. doi: 10.1177/27536130251407685 (PMC12722657; doi:10.1177/27536130251407685)
Supplement: Supplemental Material - Teaching Cancer Survivors Coping Skills for Managing Fear of Recurrence: Insights From a Pilot Randomized Controlled Trial [file sj-pdf-1-gam-10.1177_27536130251407685.pdf]

Supplemental Table 1. Baseline Sociodemographic and Medical Characteristics (N=64)

Characteristics	Total	IN FOCUS	Usual Care
**Sociodemographic**			
Age (years), M (SD)	52.4 (13.7)	51.1 (14.7)	53.7 (12.7)
Gender (female), n (%)	53 (82.8)	26 (81.3)	27 (84.4)
Ethnicity (Hispanic), n (%)	1 (1.6)	0 (0.0)	1 (3.2)
Race*, n (%)			
Alaska Native	1 (1.6)	1 (3.1)	0 (0.0)
Asian	1 (1.6)	1 (3.1)	0 (0.0)
Black or African American	5 (7.8)	4 (12.5)	1 (3.1)
Native Hawaiian or Other Pacific	0 (0)	0 (0.0)	0 (0.0)
White	59 (92.2)	29 (90.6)	30 (93.8)
Other	3 (4.7)	2 (6.3)	1 (3.1)
Married/Living as married, n (%)	41 (64.1)	23 (71.9)	18 (56.3)
Completed college, n (%)	53 (82.8)	27 (84.4)	26 (81.3)
Employed, n (%)	38 (59.4)	16 (50.0)	22 (68.8)
Private insurance, n (%)	45 (70.3)	21 (65.6)	24 (75.0)
**Medical**			
Cancer type*, n (%)			
Breast	29 (45.3)	12 (37.5)	17 (53.1)
Hematological	11 (17.2)	6 (18.8)	5 (15.6)
Genitourinary	8 (12.5)	5 (15.6)	3 (9.4)
Gynecological	5 (7.8)	2 (6.3)	3 (9.4)
Sarcoma	5 (7.8)	3 (9.4)	2 (6.3)
Head/Neck	4 (6.3)	3 (9.4)	1 (3.1)
Melanoma	2 (3.1)	1 (3.1)	1 (3.1)
Time since diagnosis (months), M (SD)	100.2 (93.3)	106.6 (104.6)	94.5 (68.7)
Time since treatment (months), M (SD)	60.0 (60.5)	56.9 (59.8)	63.2 (61.9)
Metastatic cancer, n (%)	0 (0)	0 (0)	0 (0)
Cancer treatment type*, n (%)			
Surgery	51 (79.7)	24 (75.0)	27 (84.4)
Radiation therapy	37 (57.8)	18 (56.3)	19 (59.4)
IV chemotherapy/other IV cancer	37 (57.8)	17 (53.1)	20 (62.5)
Hormonal or endocrine therapy	17 (26.6)	8 (25.0)	9 (28.1)
Oral chemotherapy/other oral therapy	13 (20.3)	8 (25.0)	5 (15.6)
Complementary or alternative therapy	4 (6.3)	1 (3.1)	3 (9.4)
Immunotherapy	9 (14.1)	8 (25.0)	1 (3.1)
Other	8 (12.5)	6 (18.8)	2 (6.3)
Comorbid medical illness (1+), n (%)	38 (59.4)	20 (62.5)	18 (56.3)
Comorbid psychiatric illness (1+), n (%)	29 (45.3)	18 (56.3)	11 (34.4)
**Fear of Cancer Recurrence (FCR)**			
FCR (FCRI severity), M (SD)	22.9 (4.6)	23.6 (4.2)	22.2 (4.9)
FCR (clinically elevated), n (%)	40 (62.5)	22 (68.8)	18 (56.3)

Note. *Participants could select more than one response. M=Mean, SD=Standard Deviation. FCRI=Fear of Cancer Recurrence Inventory
